# Differential expression of vaginal microenvironment cytokines in relation to delivery modes and their impact on postpartum pelvic floor recovery

**DOI:** 10.3389/fgwh.2026.1754695

**Published:** 2026-04-01

**Authors:** Mengling Zhou, Peishan Li

**Affiliations:** Obstetrics and Gynecology Department, Chengdu Shuangliu District Maternal and Child Health Hospital, Chengdu, Sichuan Province, China

**Keywords:** cytokines, delivery type, pelvic floor recovery, postpartum repair, remodeling, vaginal microenvironment

## Abstract

Postpartum pelvic floor dysfunction (PPFD) is a common complication that substantially impairs women’s quality of life, and its pathogenesis is closely associated with the physiological and pathological changes induced by different modes of delivery. As a key regulator of local immunity and tissue repair, the vaginal microenvironment undergoes marked alterations during and after childbirth, particularly in cytokine expression patterns. This review synthesizes current evidence on the differential expression of cytokines-including pro-inflammatory, tissue-repair, and anti-fibrotic mediators-across vaginal delivery, cesarean section, instrumental delivery (forceps or vacuum extraction), and other special circumstances such as multiple gestation and dystocia. It also elucidates the mechanisms through which these cytokines modulate postpartum pelvic floor recovery by regulating inflammation, angiogenesis, collagen remodeling, and mucosal immunity. In addition, the clinical implications for postpartum rehabilitation and emerging research directions centered on cytokine-mediated pathways are discussed. Finally, we propose a novel “immune microenvironment–pelvic floor repair” conceptual model that underscores the potential of vaginal cytokines as biomarkers and therapeutic targets for improving postpartum pelvic floor health

## Introduction

Postpartum pelvic floor dysfunction (PPFD) encompasses a range of conditions, including urinary incontinence (UI), pelvic organ prolapse (POP), sexual dysfunction, and fecal incontinence, affecting 20%–40% of women within the first year after childbirth (1). Its prevalence varies with age, parity, and mode of delivery, with higher rates observed in women undergoing vaginal delivery (VD) compared to cesarean section (CS). PPFD not only impairs physical health but also causes significant psychological distress, social withdrawal, and reduced quality of life, placing a substantial burden on healthcare systems worldwide (2).

Mode of delivery-including spontaneous vaginal delivery (SVD), cesarean section (CS), instrumental delivery (forceps or vacuum extraction), and assisted vaginal delivery-exerts distinct effects on pelvic floor tissues. SVD stretches and, in some cases, tears pelvic floor muscles, ligaments, and connective tissues, and this injury profile triggers an inflammatory, reparative, and regenerative response. CS, while avoiding direct vaginal trauma, still introduces a surgical inflammatory milieu and alters hormonal and immunological profiles. Instrumental delivery, because of greater mechanical force, often causes more severe soft-tissue injury and more persistent local inflammation, which can complicate coordinated pelvic floor recovery (3, 4).

The pelvic floor is not a single tissue compartment. It includes the levator ani muscle complex, connective tissue and fascial supports, neurovascular structures, and the vaginal wall and mucosa. That distinction matters in this review, because cytokine signaling can have different effects across muscle, fascia, and epithelium, and the timing of repair is often tissue-specific (4, 5).

The vaginal microenvironment is a dynamic ecosystem comprising a diverse microbiota, epithelial and immune cells, and soluble factors such as cytokines. Under physiological conditions, Lactobacillus species dominate the vaginal microbiota, maintaining an acidic environment (pH 3.8–4.5) that inhibits pathogenic colonization and preserves mucosal barrier integrity (6). Pregnancy and childbirth are accompanied by marked modifications in this ecosystem, including microbial shifts, epithelial stress, and immune activation (7, 8). Cytokines, as central immune mediators, coordinate tissue repair, angiogenesis, and inflammation resolution. Recent evidence links vaginal cytokine profiles to postpartum recovery outcomes, suggesting their potential as biomarkers for predicting PPFD risk and guiding therapeutic interventions (9).

**Figure 1:**
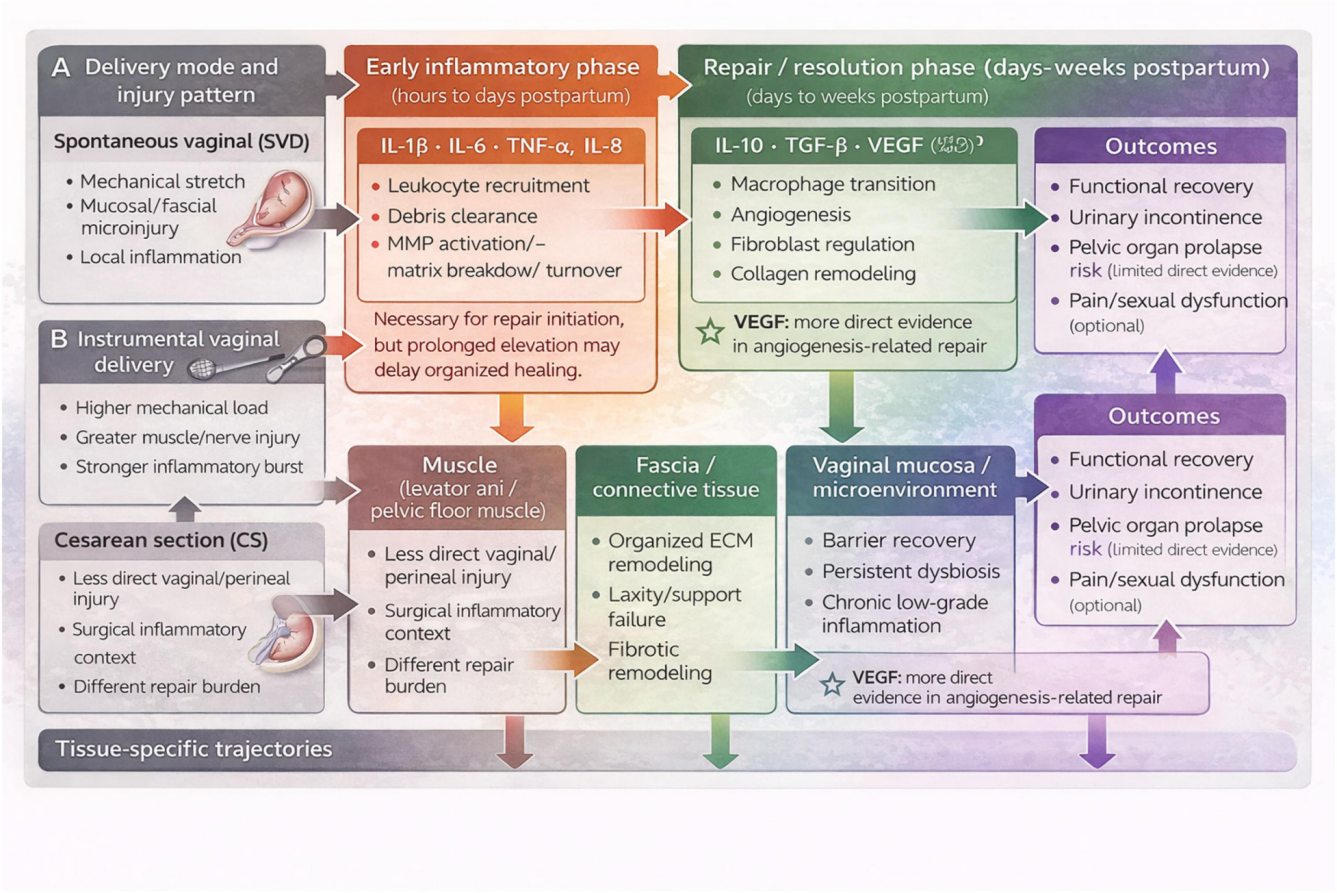
Conceptual schematic of delivery mode–associated cytokine responses, tissue-specific repair trajectories, and postpartum pelvic floor outcomes. The figure compares spontaneous vaginal delivery, instrumental vaginal delivery, and cesarean section with respect to injury pattern, early inflammatory signaling, subsequent repair/resolution pathways, and tissue-specific responses in pelvic floor muscle, fascia/connective tissue, and the vaginal mucosa/microenvironment, leading to recovery or persistent pelvic floor dysfunction.

Despite increasing interest in the vaginal microenvironment’s role in postpartum health, a comprehensive understanding of how delivery modes influence cytokine expression and subsequent pelvic floor repair remains limited. This review synthesizes current evidence on delivery mode-specific cytokine responses, elucidates their mechanistic roles in pelvic floor regeneration, and explores clinical implications for postpartum care. By integrating these findings, we aim to inform targeted strategies for the prevention and treatment of PPFD. A schematic summary of the proposed delivery mode-cytokine-repair framework is provided in Figure 1.

## Key functions of cytokines in tissue repair

Inflammatory cytokines, including interleukin-1 beta (IL-1β), tumor necrosis factor-alpha (TNF-α), and interleukin-6 (IL-6), are rapidly upregulated in response to childbirth-related tissue injury (10). These cytokines initiate the acute inflammatory response by increasing vascular permeability, recruiting neutrophils and macrophages to the injury site, and promoting the release of additional pro-inflammatory mediators. Acute inflammation is necessary for debris clearance and repair initiation, but excessive or prolonged inflammatory activity can amplify tissue damage and delay organized healing (11).

Repair-associated mediators, including transforming growth factor-beta (TGF-*β*) and vascular endothelial growth factor (VEGF), play central roles in tissue regeneration and repair-phase signaling (12). TGF-*β* promotes fibroblast proliferation and differentiation, stimulates collagen synthesis, and regulates the transition from inflammation to tissue repair (13). VEGF is a key mediator of angiogenesis and endothelial recovery, supporting oxygen delivery and stromal repair in healing tissues (14). In pelvic floor and vaginal repair, evidence supporting VEGF is more direct than that for fibroblast growth factor (FGF) or epidermal growth factor (EGF); in the postpartum vaginal setting, FGF- and EGF-related effects are best viewed as hypothesized contributors inferred from general wound-healing literature rather than defined pathways in pelvic floor dysfunction (15, 16).

Anti-fibrotic regulation, including interleukin-10 (IL-10) signaling and the matrix metalloproteinase (MMP)/tissue inhibitor of metalloproteinase (TIMP) system, helps prevent excessive fibrosis during tissue repair (17). IL-10 suppresses the production of pro-inflammatory cytokines and inhibits fibroblast activation, thereby reducing collagen deposition (18). The balance between MMPs and TIMPs is essential for collagen remodeling, as MMPs degrade aged collagen while TIMPs limit excessive degradation and support formation of functional collagen (19).

## Vaginal microenvironment and postpartum repair

The vaginal microenvironment is a highly dynamic and complex ecosystem, comprising epithelial barriers, commensal microbiota, mucosal immune cells, and a diverse array of secreted cytokines. These components cooperate to maintain mucosal integrity, mount effective immune responses, and orchestrate tissue repair-a balance that becomes especially critical following childbirth.

The vaginal epithelium, underlain by a mucosal glycocalyx and cervicovaginal fluid enriched with antimicrobial peptides and immunoglobulins, functions as both a physical and chemical barrier against pathogens (20). A healthy vaginal microbiota, typically dominated by Lactobacillus species, sustains this protective environment by producing lactic acid, bacteriocins, and hydrogen peroxide, thereby maintaining an acidic pH and inhibiting pathogen colonization. Lactic acid, in particular, can modulate mucosal immune tone and has been linked to lower expression of pro-inflammatory cytokines such as IL-6 and TNF-α in epithelial and mucosal immune contexts (6, 20). Disruption of this microbial balance-such as diminished Lactobacillus dominance postpartum-has been associated with persistent inflammation and delayed restoration of optimal microbiota composition (9).

Following delivery, acute tissue injury induces upregulation of IL-1β, IL-6, and TNF-α. These pro-inflammatory cytokines collectively mediate recruitment of neutrophils and macrophages, initiate extracellular matrix (ECM) breakdown, and trigger inflammatory cascades essential for wound debridement and microbial defense (15). As inflammation progresses, IL-10 and TGF-*β* act as key modulators to prevent excessive immune activation. These cytokines promote macrophage polarization toward reparative phenotypes, regulate fibroblast activity, and facilitate controlled matrix deposition and remodeling (21).

Tissue regeneration is further supported by growth factors, including VEGF, FGF, and EGF, which can promote angiogenesis, epithelial repair, and restoration of vascular support during mucosal healing (13, 14). Direct postpartum vaginal evidence remains limited, but VEGF is plausibly involved in early pelvic tissue repair because revascularization is required for re-epithelialization and stromal recovery; by contrast, evidence for FGF and EGF in this specific postpartum vaginal context is still largely extrapolated from broader wound-healing literature (15, 16). Structural remodeling involves a precisely balanced activity of MMPs and TIMPs (15). In pelvic floor disorders such as POP, elevated IL-6 and TNF-α levels correlate with increased MMP-1 and MMP-2 activity and reduced TIMP expression, reflecting accelerated ECM degradation and diminished tissue resilience.

## Differential expression of cytokines in vaginal microenvironment across different delivery modes

### Spontaneous vaginal delivery

SVD is typically accompanied by a rapid, transient increase in pro-inflammatory cytokines-most notably IL-1β, IL-6, interleukin-8 (IL-8), and TNF-α-within the vaginal microenvironment, maternal serum, and myometrial tissue at the onset of and during active labor (22). These mediators recruit neutrophils and monocytes, upregulate MMPs, and initiate debris clearance, angiogenesis, and fibroblast activation, all processes essential for physiological wound healing of birth-related tears and stretch injuries (9). Longitudinal studies and vaginal microbiome analyses demonstrate that this acute pro-inflammatory cytokine surge typically begins during labor and generally subsides within days postpartum as anti-inflammatory and regenerative factors-including IL-10, TGF-*β*, and VEGF-are induced to resolve inflammation and promote re-epithelialization (23). These dynamic patterns-an early pro-inflammatory burst followed by reparative signaling-have been observed across postpartum cohorts, vaginal microbiome studies, and preclinical regeneration models of pelvic floor injury (9, 23–25).

### Cesarean section

Elective CS performed in the absence of labor is associated with attenuated local vaginal immune activation compared with SVD (26). Studies report lower concentrations of several labor-associated cytokines in individuals who did not experience labor, reflecting the absence of vaginal passage, mucosal stretch, and labor-associated microbial exposures that drive local innate immune activation. In contrast, systemic inflammatory responses elicited by surgical trauma-such as incisions, anesthesia, and blood loss-can elevate serum inflammatory markers; however, this systemic pattern differs qualitatively and temporally from the labor-driven mucosal cytokine profile observed after vaginal birth. Consequently, early reparative signals within the vaginal niche-such as VEGF induction and local MMP/TIMP modulation-may be blunted after elective CS, potentially altering the tempo of ECM remodeling and pelvic-floor recovery despite minimal mechanical injury to pelvic supports (9). Notably, cesarean deliveries performed after labor or as emergencies typically display cytokine signatures resembling those of vaginal delivery, indicating that the presence and duration of labor-not the surgical intervention *per se*-largely determine local cytokine dynamics (Table 1).

**Table 1:** Differential cytokine expression in the vaginal microenvironment by delivery mode.

Delivery Mode	Elevated Cytokines	Downregulated/Lower Cytokines	Response Timing	Clinical Implications
Spontaneous Vaginal Delivery (SVD)	IL-6 IL-8 TNF-α IL-10	–	Early: 0–24 h (≤d3) Late: wk–mo	Promotes tissue repair; (9, 22, 23, 37) balanced pro-/anti-inflammatory response aids recovery
Cesarean Section (CS)	Systemic IL-1β IL-6(serum)	IL-6(vaginal) IL-8 TNF-α	Early: post-op 12–24 h (≤d3) Late: wk–mo	Lower local inflammation; (9, 26) may delay pelvic floor remodeling
Instrumental Delivery (Forceps/Vacuum)	IL-1β TNF-α IL-6 IL-8 MMP-related cytokines	–	Early: 0–24 h Prolonged: d–wk (≤2 mo*)	Strong inflammatory response; (5, 22, 27) greater pelvic trauma; higher risk of delayed recovery

SVD: Spontaneous Vaginal Delivery; CS: Cesarean Section; d = day; wk = week; mo = month. *Most often reported with significant perineal laceration.

### Instrumental delivery (forceps/vacuum extraction)

Instrumental vaginal deliveries, commonly employed during prolonged or obstructed labor, result in greater mechanical trauma to pelvic soft tissues-including the levator ani, perineal muscles, and connective tissue-and a more intense and often prolonged local inflammatory response. Observational and surgical-pathology studies associate operative vaginal delivery with increased severity of perineal tears and higher rates of muscle avulsion, conditions mechanistically linked to amplified expression of IL-1β, IL-6, and TNF-α, sustained nuclear factor-kappa B (NF-*κ*B) pathway activation, and perturbation of the MMP/TIMP balance in damaged tissues (27). This persistent inflammatory milieu can delay the transition to anti-inflammatory and regenerative programs (IL-10, TGF-*β*, angiogenic factors) and predispose to either excessive ECM degradation—weakening pelvic supports—or aberrant fibrosis, characterized by stiff, non-functional scarring. Both pathways plausibly contribute to inferior long-term pelvic-floor outcomes following instrumental delivery. Recent clinical series continue to report higher short- and long-term pelvic morbidity after forceps or vacuum-assisted deliveries compared with uncomplicated SVD, supporting a mechanistic role for delivery-associated cytokine dysregulation in postoperative tissue remodeling (22).

### Cytokine network and pelvic floor repair mechanism

Pelvic floor repair involves a complex network of cytokines that orchestrate the sequential phases of tissue healing. Understanding these cytokine networks is critical for elucidating the mechanisms underlying pelvic floor disorders and for developing targeted therapeutic strategies. This section reviews the roles of cytokines during the inflammatory, resolution, remodeling, and pathological repair phases of pelvic floor tissue regeneration.

### Inflammation phase

The inflammation phase represents the initial response to tissue injury and is characterized by activation of the innate immune system and release of pro-inflammatory cytokines. In pelvic floor repair, cytokines such as interleukin-1 (IL-1), TNF-α, and interferon-gamma (IFN-*γ*) are upregulated in response to mechanical stress or injury. These cytokines recruit immune cells-including neutrophils and macrophages-to the injury site, initiating an inflammatory response necessary for pathogen clearance and tissue debridement. However, excessive or prolonged inflammation can cause tissue damage and fibrosis, underscoring the need for tightly regulated cytokine signaling. Preclinical postpartum models also support a timed shift from pro-inflammatory to reparative signaling in pelvic floor muscle, with histologic regeneration emerging as inflammatory markers decline (25, 28). Studies indicate that cytokines such as IL-6, IL-10, and TGF-*β*, which participate in inflammation, remodeling, and repair, have dual roles in POP and UI (15, 16). They can promote tissue healing and regeneration but also exacerbate inflammation and fibrosis, contributing to disease progression. Understanding these dual roles may inform strategies to optimize the vaginal microenvironment and improve outcomes in POP and UI management (28).

### Resolution phase

Following the inflammatory response, the resolution phase is essential for terminating inflammation and initiating tissue repair. Specialized pro-resolving mediators (SPMs), including lipoxins and resolvins, counteract pro-inflammatory signals and facilitate clearance of apoptotic cells. In the pelvic floor, these mediators promote the transition from inflammation to repair, ensuring healing occurs without excessive fibrosis or scarring. SPMs inhibit neutrophil and monocyte infiltration into inflamed tissues, promote apoptosis of pro-inflammatory lymphocytes, and stimulate reparative responses in fibroblasts, endothelial cells, and other tissue-resident cells. Collectively, these actions restore tissue homeostasis and support optimal recovery (29, 30).

### Remodeling phase

The remodeling phase encompasses maturation and reorganization of the ECM, restoring tissue integrity and function. Cytokines such as TGF-*β* and IL-10 are key regulators, promoting collagen synthesis and fibroblast differentiation into myofibroblasts (31). These processes are vital for strengthening pelvic floor tissues. Dysregulation of cytokine signaling during remodeling, however, can result in pathological fibrosis or insufficient tissue strength, contributing to conditions such as POP. Macrophage responses to polypropylene mesh implants demonstrate that biomaterial properties can modulate inflammatory responses and influence healing outcomes, highlighting the importance of both biological and material factors during remodeling (32).

### Pathological repair

Pathological repair occurs when normal healing is disrupted, resulting in aberrant tissue remodeling and impaired function. In the pelvic floor, this phase is often characterized by chronic inflammation, excessive collagen deposition, and fibrosis (16). Cytokines such as IL-6 and IL-10 have been implicated in these processes, with elevated levels observed in the vaginal wall of patients with POP. Profiling cytokine dynamics in pathological repair can guide therapeutic strategies aimed at modulating these pathways to promote normal tissue healing and prevent recurrence of pelvic floor disorders (30). Experimental studies demonstrate that exosomes derived from TNF-α-treated bone marrow stem cells can alleviate pelvic floor dysfunction in rats, indicating potential translational applications for cytokine-targeted therapies in pathological repair (33).

### Clinical and rehabilitation significance

Evidence indicates that the mode of delivery significantly influences early postpartum pelvic floor muscle function. Vaginal deliveries-particularly forceps-assisted-are associated with less favorable pelvic floor electromyographic patterns, including reduced activation amplitude and impaired recruitment in some cohorts, together with higher rates of urinary incontinence compared with cesarean sections (5, 34–36). Complementary animal studies demonstrate that expression of C-C motif chemokine ligand 7 (CCL-7) in pelvic tissues is reduced following cesarean delivery relative to vaginal birth, suggesting that CCL-7 may serve as a biomarker of tissue injury and future pelvic floor dysfunction (PFD) risk (37). Integrating cytokine profiling, including peripartum CCL-7 and other inflammatory markers, could help identify high-risk individuals and personalize delivery method selection to optimize postpartum pelvic floor outcomes.

Monitoring cytokine dynamics postpartum may also predict recovery trajectories and guide tailored interventions. For example, manual lymphatic drainage combined with abdominal breathing significantly reduces inflammatory cytokines IL-6 and TNF-α in women with postpartum perineal edema, correlating with faster clinical recovery (38). Molecular analyses in POP and UI further emphasize the dual roles of cytokines such as IL-6, IL-10, and TGF-*β*, which can promote both tissue repair and pathological fibrosis depending on context (15). Tracking shifts in pro- versus anti-inflammatory cytokines may provide prognostic markers: persistent elevation of IL-6 or TNF-α may indicate delayed recovery or excessive scarring, whereas early upregulation of IL-10 or TGF-*β* may predict favorable tissue remodeling and functional outcomes.

Targeted modulation of cytokine pathways presents novel therapeutic opportunities. Preclinical and early clinical studies indicate that pelvic floor electrical stimulation increases TGF-*β* expression, thereby promoting tissue repair (15). Conversely, pro-inflammatory cytokine inhibitors-including those incorporated in polypropylene meshes-demonstrate potential in reducing chronic inflammation in POP models. These findings suggest that locally applied IL-10 analogs, TGF-*β* inhibitors, or physical stimulation techniques could strategically regulate cytokine networks and enhance functional tissue remodeling. Restoration of vaginal microbiota balance may further support pelvic floor recovery. In a postpartum cohort, reduced Lactobacillus abundance and increased microbial diversity correlated with pelvic floor muscle weakness and urinary incontinence (39). Probiotic interventions aimed at restoring Lactobacillus dominance may mitigate the inflammatory milieu and favor tissue healing, although direct clinical trials in PFD contexts are still lacking. Physical rehabilitation remains a cornerstone of postpartum recovery. Survey data reveal that many postpartum individuals lack guidance on pelvic floor rehabilitation, despite the demonstrated efficacy of such therapies in symptom relief (40).

Figure 1 Cytokine-centered framework linking delivery mode, vaginal microenvironment, and postpartum pelvic floor repair. Delivery mode determines the pattern and magnitude of tissue loading, local inflammation, and downstream repair signaling after childbirth. SVD is typically associated with mechanical stretch and mucosal/fascial microinjury, instrumental vaginal delivery with greater neuromuscular and connective tissue trauma, and cesarean section with a distinct surgical inflammatory context. The early phase is dominated by IL-1β, IL-6, TNF-α, and related mediators that support leukocyte recruitment and matrix turnover; the later repair/resolution phase is shaped by IL-10, TGF-*β*, VEGF, and rebalancing of ECM remodeling pathways. Tissue-level outcomes differ across pelvic floor muscle, fascia/connective tissue, and vaginal mucosa, and these trajectories can culminate in either functional recovery or persistent dysfunction, including urinary incontinence and pelvic organ prolapse risk.

## Conclusion

The vaginal microenvironment undergoes profound immunological and structural changes in response to childbirth, with cytokine signaling playing a central role in orchestrating pelvic floor repair. Distinct cytokine expression patterns associated with different delivery modes may influence tissue recovery trajectories, underscoring their potential as both biomarkers and therapeutic targets. Advances in multi-omics technologies and immunomodulatory interventions are paving the way for precision postpartum care. Integration of cytokine profiling into clinical practice could inform individualized delivery mode selection, predict PFD risk, and optimize recovery through targeted therapeutic strategies. Ultimately, translating these insights into evidence-based guidelines has the potential to improve maternal health outcomes and reduce the burden of pelvic floor disorders.
